# Impact of familial risk factors on management and survival of early-onset breast cancer: a population-based study

**DOI:** 10.1038/sj.bjc.6602914

**Published:** 2005-12-13

**Authors:** H M Verkooijen, P O Chappuis, E Rapiti, G Vlastos, G Fioretta, S Sarp, A P Sappino, H Schubert, C Bouchardy

**Affiliations:** 1 Geneva Cancer Registry, Institute for Social and Preventive Medicine, Geneva University, Switzerland; 2 Division of Oncology, Department of Internal Medicine, Geneva University Hospitals, Switzerland; 3 Division of Medical Genetics, Department of Gynecology and Obstetrics, Geneva University Hospitals, Switzerland; 4 Unit of Senology and Gynecologic Oncology, Department of Gynecology and Obstetrics, Geneva University Hospitals, Switzerland

**Keywords:** breast cancer, population-based, treatment, survival, family history

## Abstract

This population-based study evaluates the impact of a strong family history of breast cancer on management and survival of women with early-onset disease. We identified all breast cancer patients ⩽50 years, recorded between 1990 and 2001 at the Geneva familial breast cancer registry. We compared patients at high familial risk and low familial risk in terms of tumour characteristics, method of detection, treatment, survival and breast cancer mortality risk. Compared to patients at low familial risk (*n*=575), those at high familial risk (*n*=58) received significantly more often systemic therapy, especially for node-negative or receptor-positive disease. Five-year disease-specific survival rates of patients at high *vs* low familial risk were 86 and 90%, respectively. After adjustment, there was no difference in breast cancer mortality in general. A strong family history nonsignificantly increased breast cancer mortality in patients ⩽40 years (adjusted hazard ratio (HR) 4.0, 95% CI 0.8–19.7) and in patients treated without chemotherapy (adjusted HR 2.7, 95% CI 0.6–12.5). A strong family history of breast cancer is associated with an increased use of systemic therapy in early-onset patients. Although a strong family history does not seem to affect survival in general, it may impair survival of very young patients and patients treated without adjuvant chemotherapy. Owing to the limited number of patients in this study, these results should be used only to generate hypotheses.

One of the most important risk factors for breast cancer is the occurrence of breast or ovarian cancer among family members. Women with one or more first-degree relatives with breast cancer have a 1.8–3.0-fold increased risk of developing the disease (Pharoah *et al*, 1997; Collaborative Group on Hormonal Factors in Breast Cancer, 2001). Nevertheless, only about 5–10% of breast cancer patients carry a genetic predisposition to breast and/or ovarian cancer due to a highly penetrant germline mutation (Narod and Foulkes, 2004).

Some asymptomatic women carrying germline mutations in *BRCA1* or *BRCA2* genes undergo prophylactic bilateral mastectomy and/or salpingo-oophorectomy to reduce their risk of developing breast and/or ovarian cancer (Meijers-Heijboer *et al*, 2001; Stefanek *et al*, 2001; Rebbeck *et al*, 2002). Others are offered periodic clinical examination and breast imaging (mammography, ultrasound and magnetic resonance imaging). For unaffected women with an unknown or noninformative mutation status, but with a strong family history of breast cancer, intensive surveillance is also highly recommended (Pichert *et al*, 2003), as it may lead to earlier detection of breast cancer in a more favourable stage (Eccles *et al*, 2000; Tilanus-Linthorst *et al*, 2000).

Treatment guidelines of breast cancer occurring in *BRCA1/BRCA2* mutation carriers or in patients with an increased familial risk are not well established. The risk of local recurrence does not appear to be higher among patients with a strong family history than among low familial risk patients, rendering high familial risk patients equally eligible for breast-conserving surgery (Eccles *et al*, 2001; Vlastos *et al*, 2002; Robson *et al*, 2004). However, their risk for contralateral disease is highly increased (Robson *et al*, 2004).

Conflicting data exist on the impact of a family history of breast or ovarian cancer on the outcome of breast cancer (Ruder *et al*, 1988; Chappuis *et al*, 1999; Russo *et al*, 2002). Some studies have shown improved survival rates among breast cancer patients with affected relatives compared to those without a family history (Malone *et al*, 1996; Mohammed *et al*, 1998), some reported poorer survival rates (Slattery *et al*, 1993; Gonzalez-Angulo *et al*, 2005), and others did not find any survival difference between patients with or without a family history (Greenberg *et al*, 1985; Kinoshita *et al*, 2004). Although most of these studies used multivariate analysis to adjust mortality risks for other prognostic variables, none of them adjusted for use of systemic therapy.

In this study, we focus on young breast cancer patients. We will show that, among women with early-onset breast cancer, the presence of a strong family history does not lead to earlier diagnosis of the disease. However, family history has a strong impact on the use of systematic therapy. In addition, we will show that women with a strong family history have similar breast cancer survival rates as patients with a low familial risk, except for certain subgroups.

## METHODS

We used information from the Geneva cancer registry, which records all incident cancers occurring in the population of the Geneva canton (approximately 420 000 inhabitants) since 1970. It collects information from various sources and is considered accurate, as attested by its very low percentage (<2%) of cases recorded from death certificates only (Bouchardy, 1997). All hospitals, pathology laboratories, and private practitioners in the canton are requested to report all cancer cases. Trained tumour registrars systematically abstract data from medical and laboratory records. Physicians regularly receive enquiry forms to complete missing clinical and therapeutic data. Recorded data include sociodemographic information, method of diagnosis, type of confirmation, tumour characteristics coded according to the International Classification of Diseases for Oncology (World Health Organization, 1976), stage of disease at diagnosis, hormonal receptor status and treatment during the first 6 months after diagnosis. The registry regularly assesses survival, taking as reference date the date of confirmation of diagnosis or the date of hospitalisation (if it preceded the diagnosis and was related to the disease). In addition to passive follow-up (standard examination of death certificates and hospital records), active follow-up is performed yearly using the files of the Cantonal Population Office (office in charge of the registration of the resident population). Cause of death is taken from clinical files.

In 1999, the Geneva Cancer Registry set up a Familial Breast Cancer Registry, by extending its data set to the detailed family history of cancer for all women diagnosed with invasive breast cancer in the Geneva population (Bouchardy *et al*, 2002). For breast cancer patients diagnosed between 1990 and 1999, family history was collected retrospectively, using information from medical records from the public university hospitals and private physicians. For 90% of the breast cancer patients, information on family history was obtained and the accuracy of this retrospectively retrieved information has been validated (Murff *et al*, 2004; Verkooijen *et al*, 2004). Since January 2000, family history on ⩾3 generations is prospectively collected by sending standard questionnaires to health-care providers.

For the purpose of this study, we classified breast cancer patients into three familial risk categories according to the number of relatives diagnosed with breast or ovarian cancer, their age at diagnosis and their degree of kinship (Hampel *et al*, 2004). The *low familial risk category* included breast cancer patients without first- or second-degree relatives with breast or ovarian cancer (i.e. sporadic cases). The *high familial risk category* included patients who reported one of the following family histories: (1) ⩾1 first-degree relative with breast or ovarian cancer ⩽50 years; (2) ⩾2 first-degree relatives with breast/ovarian cancer at any age; (3) ⩾3 cases of breast/ovarian cancer among first- or second-degree relatives. Patients with other types of family history were classified into the *moderate familial risk category.*

We restricted the current study to women with early-onset breast cancer (⩽50 years). We excluded patients with a moderately increased familial risk in order to maximise the difference in effect of family history. We divided age into two categories (⩽40 years, 41–50 years). We categorised method of detection as due to symptoms (i.e. palpable lump, nipple discharge, etc.), fortuitously (i.e. during the work-up or treatment of another unrelated illness), breast self-examination and surveillance (i.e. screening of healthy individuals by means of physical examination, mammography, ultrasound or MRI).

Socioeconomic status was based on the woman's last occupation or, for the unemployed, that of the spouse. Four levels were considered as follows: low (manual employees, skilled and unskilled workers), middle (non-manual employees and administrative staff), high (professionals, executives, administrators) and unknown. For staging, we used the pathological pTNM (tumour node metastasis) classification system or, when not available, the clinical cTNM classification (Sobin and Wittekind, 2002). Tumours were classified as T1 (⩽2 cm), T2 (2–5 cm), T3 (>5 cm), T4 (invasion to chest wall/skin and inflammatory carcinoma) and TX (unknown). Axillary lymph node invasion was classified as N0 (no invasion), or N+ (including N1 – movable – , N2 – fixed – and NX – unknown). Distant metastasis was classified as M0 (absent), M1 (present) or MX (unknown). Stage was classified in five groups: stage I (Tis or T1 and N0), stage II (T0 or T1 and N1, T2 and N0 or N1, T3 and N0), stage III (T0 or T1 or T2 and N2, T3 and N1 or N2, T4 and any N, any T and N3), stage IV (M1) and unknown. Tumor size was categorised as <1, 1–1.9, 2–4.9 and ⩾5 cm. Tumour differentiation (grade) was classified as well differentiated (grade 1), moderately differentiated (grade 2) and poorly differentiated (grade 3). Oestrogen receptor status was determined by standard immunohistochemical reaction and considered positive when ⩾10% of the cancer cells expressed oestrogen receptors.

Locoregional therapy was categorised as breast-conserving surgery followed by radiotherapy, mastectomy, bilateral mastectomy (defined as amputation of both breasts for unilateral breast cancer) and other (including tumorectomy without radiotherapy, no surgery). Use of chemotherapy and hormonotherapy was categorised as yes *vs* no. Contralateral breast cancer was categorised as ‘synchronous’ if detected within 6 months after diagnosis, and ‘metachronous’ if diagnosed later. Information on *BRCA1/BRCA2* status was not routinely available.

### Statistics

To compare method of discovery, stage at diagnosis and treatment between patients at high and low familial risk, we used a case–control approach. Cases were breast cancer patients with a strong family history and controls were patients without affected relatives. With unconditional univariate logistic regression analysis, we identified the factors that were significantly associated with a high familial risk. With multivariate analyses, we adjusted the relative risks for all factors significantly associated with family history in univariate analysis.

We used Kaplan–Meier analysis to calculate breast cancer survival rates for women at high familial risk and those at low familial risk. With univariate Cox proportional hazards analysis, we identified all variables significantly linked to survival. Finally, we calculated breast cancer mortality risk (hazard ratio, HR) for patients with a highly increased familial risk compared to those without a family history, adjusting for all other factors significantly linked to survival. To evaluate if the effect of familial risk was different between age groups, we stratified by different age categories (<41 years, 41–50 years) and tested for interaction. We also investigated if the use of chemotherapy changed the effect of a strong family history. Data were analysed with SPSS software and differences were considered significant at a two-sided *P*-value <0.05.

## RESULTS

Between 1990 and 2001, 3709 women were diagnosed with invasive breast cancer in the canton of Geneva. Among them, 824 (22%) were ⩽50 years of age. A total of 58 (7%) patients reported a family history classified as high familial risk, 191 (23%) a moderate familial risk and 575 (70%) reported no first- or second-degree relatives with breast or ovarian cancer. Patients with a moderate familial risk were excluded from further analysis. Details on family history of the patients in the high familial risk category are presented in Table 1.

In Table 2, we present the patient and tumour characteristics of breast cancer patients with high *vs* low familial risk. There was no significant difference in age or period of diagnosis. Women with a strong family history had less frequently stage I disease at diagnosis (24 *vs* 36%, *P*=0.043), and more often stage III disease (19 *vs* 9%, *P*=0.014), when compared to low familial risk patients. After adjustment for age, women at high familial risk were significantly more likely to be diagnosed with stage III disease than patients without a family history of breast cancer (adjusted odds ratio (OR) 3.3, 95% CI 1.4–7.8).

Women at high familial risk tended to have more frequently axillary lymph node involvement compared to women at low familial risk (53 *vs* 43%, respectively), grade 1 tumours (33 *vs* 23%, respectively) and oestrogen receptor-positive tumours (74 *vs* 64%, respectively), but these differences were not statistically significant.

The risk of presenting synchronous bilateral breast cancer was increased among patients with a high familial risk (adjusted OR 3.9, 95% CI 1.0–15.8, *P*=0.051), while the risk to develop metachronous contralateral breast cancer was similar for both groups (median follow-up 5.2 years).

There were no differences in histological subtypes between the high familial risk and low familial risk groups (data not shown): 81% of low familial risk patients *vs* 85% of high familial risk patients had ductal histology, 10% of low and high familial risk patients lobular histology and 2% of low familial risk *vs* 0% of high familial risk patients had medullar histology.

Table 3 shows the methods of tumour detection and management of breast cancer according to familial risk. No significant differences were observed between the high and low familial risk patients. The proportion of tumours detected by surveillance was slightly higher among women at high familial risk than those at low familial risk (28 *vs* 23%, respectively), but this result was not significant.

Breast-conserving therapy was performed at a similar rate in patients at high *vs* low familial risk. Bilateral mastectomy for unilateral disease was more common among women with a strong family history (adjusted OR 4.2, 95% CI 0.9–18.4).

Patients at high familial risk received more often chemotherapy than low familial risk patients (74 *vs* 65%, respectively), but this difference was not significant. A higher proportion of women at high familial risk received hormonotherapy (57 *vs* 43% in low familial risk women, *P*=0.013). After adjustment for age, stage, oestrogen receptor status, locoregional therapy and chemotherapy, the probability to receive hormonotherapy remained significantly increased among patients at high familial risk (adjusted OR 1.9, 95% CI: 1.1–3.4).

Table 4 shows the use of systemic therapy according to axillary lymph node invasion and oestrogen receptor status. In the group of patients with node-negative disease, those at high familial risk were more likely to be treated with any type of systemic therapy (88 *vs* 65% of low risk women, *P*=0.024). The proportion of women treated with hormonotherapy was also significantly higher (67 *vs* 39%, *P*=0.007), as was the proportion of women treated with both hormonotherapy and chemotherapy (38 *vs* 19%, *P*=0.034). In the group of lymph node-positive patients, high familial risk patients received equally frequently systemic therapy as low familial risk patients (94 *vs* 93%, respectively), but they were more often treated with both chemotherapy and hormonotherapy (52 *vs* 40%, respectively, *P*=0.011).

In the group of women with oestrogen receptor-positive tumours, patients at high familial risk received significantly more frequently hormonotherapy when compared to patients at low familial risk (74 *vs* 56%, respectively, *P*=0.020), chemotherapy (77 *vs* 61%, respectively, *P*=0.044) and both (58 *vs* 38%, respectively, *P*=0.011). In the group of patients with oestrogen receptor-negative tumours, no significant differences in use of systemic therapy were observed.

**Figure 1:**
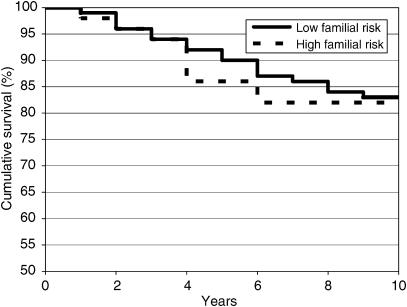
Breast cancer-specific survival for young women (⩽50 years) at highly increased familial risk and women at low familial risk.

Figure 1 shows the survival rates of women with a high familial risk and those at low familial risk. At 5 years, the breast cancer-specific survival of women at high familial risk was 86% (95% CI: 75–97%) compared to 90% (95% CI: 87–93%) for women without a family history. At 10 years, survival rates were 82% (95% CI: 69–95%) and 82% (95% CI: 79–87%), respectively.

In univariate analysis, well-known prognostic variables such as socioeconomic status, stage, histological grade, oestrogen receptor status, locoregional treatment and chemotherapy proved to be significantly related to breast cancer-specific survival (data not shown). Use of hormonotherapy decreased the breast cancer mortality risk by 40% (HR 0.6, 95% CI: 0.4–1.1), but this result was not statistically significant. In univariate analysis, patients with a strong family history did not have an increased risk of dying of breast cancer compared to patients without a family history (HR 1.1, 95% CI: 0.5–2.2). After adjustment for all factors significantly linked to survival in univariate analysis (including hormonotherapy), the risk of dying of breast cancer was not significantly different among women at high familial risk compared to those at low familial risk (adjusted HR 1.3, 95% CI: 0.6–2.8).

Table 5 presents the breast cancer mortality risks of patients with a high familial risk compared to those at low familial risk stratified by age and use of chemotherapy. For women aged 41–50 years, a highly increased familial risk was not associated with an increased risk to die of breast cancer (HR 1.0, 95% CI: 0.4–2.6). However, in the category of patients of 40 years or younger, breast cancer mortality risk was increased for patients at high familial risk, although not significantly (HR 4.0, 95% CI: 0.8–19.7). Test for interaction between age and familial risk was also not significant.

In the subgroup of patients treated with chemotherapy, a strong family history was not associated with an increased breast cancer mortality risk (HR 1.0, 95% CI: 0.4–2.6) (Table 5). Among patients treated without chemotherapy, a strong family history increased the breast cancer mortality risk, although not significantly (HR 2.7 95% CI: 0.6–12.5). Test for interaction between use of chemotherapy and familial risk was not significant either.

Of the 10 high familial risk women younger than 41 years, two did not receive systemic therapy. Both of them died within 5 years after diagnosis.

In the subgroup of patients with poorly differentiated (grade 3) tumours, a strong family history was not associated with a significantly increased mortality risk (adjusted HR 1.2, 95% CI 0.4–4.0) (data not shown).

Finally, we analysed the survival of the 191 patients at moderate familial risk. Their 5 and 10 years specific survival rates were 93% (95% CI 89–97%) and 85% (95% CI 77–93%), respectively, and not significantly different than those of low familial risk patients. Also, the multiadjusted breast cancer mortality risk of patients with a moderately increased familial risk was not significantly different than that of patients at low familial risk (HR 0.7, 95% CI 0.4–1.3).

## DISCUSSION

In this population-based study, we show that breast tumours occurring at an early age among women belonging to high-risk families were neither discovered more frequently by screening or surveillance nor diagnosed at an earlier stage than in patients without a family history of breast cancer. However, after the diagnosis of invasive breast cancer, patients at high familial risk were treated differently compared to patients without a family history, particularly regarding the prescription of systemic therapy. In addition, this study shows that a strong family history of breast and/or ovarian cancer was not associated with a decreased survival after early-onset breast cancer, except, maybe, for very young patients and patients not receiving chemotherapy.

Standard protocols for the surveillance of women carrying *BRCA1/BRCA2* germline mutations become increasingly widespread, but vary from one centre to another (Stefanek *et al*, 2001; Eisinger *et al*, 2004; Warner *et al*, 2004). For women with noninformative genetic test results and/or a positive family history, some screening protocols have been proposed (Pichert *et al*, 2003). These protocols could lead to an earlier diagnosis of breast cancer at even preclinical stages (Tilanus-Linthorst *et al*, 2000). We were surprised to find that in Geneva, young breast cancer patients with a strong family history were not diagnosed more frequently by targeted screening. Moreover, patients with a strong family history had less often early-stage disease at diagnosis and more often locally advanced disease than early-onset patients with no family history. The absence of association between a positive family history and earlier detection of the disease has previously been reported (Groenendijk *et al*, 2002; Madlensky *et al*, 2005). One explanation could be that, compared to sporadic cases, *BRCA1/BRCA2*-related breast cancers, as well as breast cancers diagnosed in the context of a strong family history, are less susceptible to be detected by mammography screening (Brekelmans *et al*, 2001; Stoutjesdijk *et al*, 2001). Also, some of the high familial risk patients might have had fast growing cancers, which became symptomatic in between two screening interventions. Another reason could be unawareness of the public and professionals on the importance of a strong family history as a major risk factor for breast cancer.

This study is the first one to investigate the impact of family history on the management of early-onset breast cancer in a population-based setting. Despite the limited number of patients with a strong family history, we were able to demonstrate important and significant differences in the management of patients at high *vs* low familial risk. After adjustment for age, stage at diagnosis, oestrogen receptor status and use of chemotherapy, early-onset breast cancer patients with a high familial risk were twice as likely to be treated with systemic therapy, especially for lymph node-negative and for oestrogen receptor-positive disease.

This study gives no insight into the reasons why patients at high familial risk received more frequently systemic treatments. One can hypothesise that breast cancer occurring in the context of a strong family history may be considered as a more aggressive disease. Although some clear histopathological differences have been observed between *BRCA1-*related breast cancer and sporadic cases (more high grade, oestrogen receptor-negative and p53-positive tumours in *BRCA1* mutation carriers), this has not been demonstrated for familial cases of breast cancer (Breast Cancer Linkage Consortium, 1997; van de Vijver, 1999). We did not observe a significant difference in proportion of grade 3 or oestrogen receptor-negative tumours between women at high *vs* low familial risk, but the proportion of stage III disease was significantly higher. However, differences in systemic treatment persisted after adjustment for stage.

Another explanation for the more complete treatment of women at high familial risk could be the patient's attitude towards her disease and treatment options. Women at high familial risk have usually witnessed the disease in family members and may even have lost a close relative from breast cancer. Such a personal experience might result in a willingness to accept or demand more aggressive or complete therapy. Until now, however, there are no solid data to confirm differences in attitude towards breast cancer treatment among women at high *vs* low familial risk.

Finally, one can hypothesise that treating physicians consider women with a strong family history at increased risk for local recurrence, contralateral disease and, ultimately, death from breast cancer compared to patients without a family history. By giving systemic therapy or proposing the option of preventive bilateral mastectomy, physicians expect to reduce these risks. In the 1990s, several multicentric trials were initiated to determine the efficacy of tamoxifen as a chemopreventive drug to reduce breast cancer incidence among moderate- and high-risk women (Jordan, 1997; Osborne, 1999; Cuzick *et al*, 2002). Although none of the Geneva hospitals participated in these trials, the publicity around this topic might have influenced the attitude of the local physicians. This does not explain the increased use of chemotherapy, but may have increased the use of hormonotherapy among high familial risk patients.

Differences in use of systemic therapy between young women with a strong family history *vs* those without affected relatives can have important impact on breast cancer mortality. Since the late 1990s, several studies have demonstrated the importance of adjuvant chemotherapy among young breast cancer patients by showing that the unfavourable impact of young age on breast cancer survival is valid only among patients treated without chemotherapy, and that among women treated with chemotherapy, young age is no longer an independent prognostic risk factor (Greenberg *et al*, 1985; Kroman *et al*, 2000; Rapiti *et al*, 2005). In this study, we show that young breast cancer patients without axillary lymph node involvement were more likely to be treated with chemotherapy, hormonotherapy or both, if they had a strong family history of breast or ovarian cancer. If, in other series as well, more complete treatment would have been given to high familial risk patients, the impact of a strong family history on survival could have been underestimated. This could explain why, in some studies, women with a strong family history survive better than women without affected relatives.

To our knowledge, it is the first time that use of systemic therapy was accounted for while estimating the impact of a strong family history on survival after early-onset breast cancer. Despite the absence of statistically significant results, due to the low power of our study, our data suggest that accounting for treatment is important. Especially in the subgroup of women who did not receive chemotherapy, the presence of a positive family history appeared to increase the risk to die of breast cancer. This observation is substantiated by two earlier studies among Ashkenazi Jewish breast cancer patients. In these studies, *BRCA1* status was a strong predictor of breast cancer survival only among women who did not receive chemotherapy, while among women who received chemotherapy it was not (Goffin *et al*, 2003; Robson *et al*, 2004).

The results of our study also suggest that among very young women (under 41 years of age at diagnosis), a strong family history might have a negative impact on breast cancer survival. These results are substantiated by a recent study from investigators at the MD Anderson Cancer Center, showing that among very young breast cancer patients (⩽35 years), a family history of ovarian cancer strongly impaired disease-free and overall survival (Gonzalez-Angulo *et al*, 2005). Nevertheless, the low number of cases prevented us from drawing definite conclusions and the results should be used only to generate hypotheses. A possible explanation might be that the proportion of *BRCA*1 carriers is higher among very young women at high familial risk. As *BRCA*1-related tumours exhibit poor prognostic characteristics (negative oestrogen receptor status and high grade) and have often been shown to be associated with impaired survival rates (Moller *et al*, 2002), this could explain the negative impact of strong family history in the subgroup of very young women. However, in our group of patients, tumours of the ‘younger’ patients with a strong family history were neither of higher grade, nor more often oestrogen receptor negative than the tumours of the ‘older’ patients with a strong family history. Therefore, the effect of a strong family history on survival of very young women needs further investigation.

Based on this study, we conclude that guidelines on screening and management of young women at high familial risk are needed. By better informing the public and primary care physicians on the importance of family history, it should be possible to increase the number of women identified as high risk of developing breast cancer and to propose to these women specific screening and prevention protocols. Finally, additional research is needed to confirm if a strong family history of breast cancer impairs survival of very young patients and those treated without chemotherapy.

## Figures and Tables

**Table 1:** Description of family history of breast and ovarian cancer among 58 breast cancer patients diagnosed ⩽50 years and classified as high familial risk

**Family history**	***n*=58**
*At least one FDR with breast/ovarian cancer ⩽50 years*	*30*
One FDR ⩽50 years with breast cancer (with or without other relatives with breast cancer)	26
One FDR ⩽50 years with bilateral breast cancer	4
	
*At least two breast/ovarian cancers among FDRs*	*4*
Two FDRs with breast cancer	2
One FDR with bilateral breast cancer and one SDR with breast cancer	2
	
*At least three cases of breast/ovarian cancer among FDRs or SDRs*	*24*
Two FDRs with ovarian cancer and one FDR with breast cancer	1
One FDR with bilateral breast cancer, two SDRs with breast cancer and one SDR with ovarian cancer	1
Two relatives with breast cancer and one with ovarian cancer	2
Three relatives with breast cancer	15
Four relatives with breast cancer	3
Five relatives with breast cancer and one with ovarian cancer	1
Six relatives with breast cancer	1

Geneva Cancer Registry 1990–2001.

FDR=first-degree relative: mother, sister(s), daughter(s).

SDR=second-degree relative: grandmother(s), aunt(s), niece(s).

**Table 2:** Patient and tumour characteristics for breast cancer patients at high and low familial risk

	**High familial risk (*n*=58)**	**Low familial risk (*n*=575)**	**Unadjusted OR (95% CI)**	**Age- and stage-adjusted OR (95% CI)**
*Age category*				
41–50 years	48 (82%)	441 (77%)	1^a^	1^a^^,^^b^
⩽40 years	10 (18%)	134 (24%)	0.7 (0.3–1.4)	0.7 (0.3–1.4)
				
*Period of diagnosis*				
1990–1993	13 (22%)	163 (28%)	1^a^	1^a^
1994–1997	20 (35%)	201 (35%)	1.2 (0.6–2.6)	1.3 (0.6–2.8)
1998–2001	25 (43%)	211 (37%)	1.5 (0.7–3.0)	1.6 (0.8–3.3)
				
*Stage*				
Stage I	14 (24%)	208 (36%)	1^a^	1^a^^,^^c^
Stage II	28 (48%)	272 (47%)	1.5 (0.8–3.0)	1.5 (0.8–3.0)
Stage III	11 (19%)	49 (9%)	3.3 (1.4–7.8)**	3.3 (1.4–7.8)**
Stage IV	1 (2%)	30 (5%)	0.5 (0.1–3.9)	0.5 (0.1–3.9)
Unknown	4 (7%)	16 (3%)	—	—
				
*Tumour size*				
<1 cm	13 (22%)	89 (16%)	1^a^	1^a^^,^^d^
1–1.9 cm	16 (28%)	224 (39%)	0.5 (0.2–1.1)	0.4 (0.2–1.0)
2–4.9 cm	16 (28%)	166 (29%)	0.7 (0.3–1.4)	0.6 (0.3–1.3)
>5 cm	4 (7%)	21 (4%)	1.3 (0.4–4.4)	1.0 (0.3–3.6)
Unknown	9 (16%)	75 (13%)	—	—
				
*Axillary lymph node status*				
Negative	24 (41%)	311 (54%)	1^a^	1^a^^,^^e^
Positive	31 (53%)	248 (43%)	1.6 (0.9–2.8)	1.6 (0.9–3.0)
Unknown	3 (5%)	16 (3%)	—	—
				
*Histologic grade*				
1	19 (33%)	130 (23%)	1^a^	1^a^
2	17 (29%)	217 (38%)	0.5 (0.3–1.1)	0.5 (0.2–1.0)
3	17 (29%)	171 (30%)	0.7 (0.3–1.4)	0.6 (0.3–1.2)
Unknown	5 (9%)	57 (10%)	—	—
				
*Oestrogen receptor status*				
Positive	43 (74%)	367 (64%)	1^a^	1^a^
Negative	11 (19%)	143 (25%)	0.7 (0.3–1.3)	0.6 (0.3–1.3)
Unknown	4 (7%)	65 (11%)	—	—
				
*Bilateral breast cancer*				
No	53 (91.0%)	546 (95%)	1^a^	1^a^
Yes, synchronous	3 (5%)	9 (2%)	3.4 (0.9–13.1)	3.9 (1.0–15.8)
Yes, metachronous	2 (3%)	20 (4%)	1.0 (0.2–4.5)	1.0 (0.2–4.4)

OR=odds ratio; CI=confidence interval.

a Reference category.

b Adjusted OR for age category was not adjusted for age.

c Adjusted OR for stage was not adjusted for stage.

d OR for tumour size was adjusted for age and lymph node status (and not for stage).

e OR for lymph node status was adjusted for age and tumour size (and not for stage).

** *P*<0.01.

**Table 3:** Methods of detection and treatment of breast cancer according to family history

	**Familial risk**			
	**High familial risk (*n*=58)**	**Low familial risk (*n*=575)**	**Unadjusted OR (95% CI)**	**Multi-adjusted OR (95% CI)**
*Method of detection*				
Self examination	22 (38%)	213 (37%)	1^a^	1^a^^,^^b^
Surveillance	16 (28%)	132 (23%)	1.2 (0.6–2.3)	1.3 (0.7–2.8)
Symptoms	17 (29%)	203 (35%)	0.8 (0.4–1.6)	0.7 (0.4–1.4)
Fortuitous	3 (5%)	17 (3%)	1.7 (0.5–6.3)	1.8 (0.5–7.0)
Unknown	0 (0%)	10 (2%)	—	—
				
*Locoregional treatment*				
Breast-conserving surgery	36 (62%)	338 (59%)	1^a^^,^	1^a^^b^
Mastectomy	14 (24%)	164 (29%)	0.8 (0.4–1.5)	0.7 (0.3–1.2)
Bilateral mastectomy	3 (5%)	6 (1%)	4.7 (1.1–19.6)*	4.2 (0.9–18.4)
Other	5 (9%)	67 (12%)	0.7 (0.3–1.9)	0.6 (0.2–1.7)
				
*Chemotherapy*				
No	15 (26%)	201 (35%)	1^a^	1^a^^,^^c^
Yes	43 (74%)	374 (65%)	1.5 (0.8–2.8)	1.3 (0.6–2.7)
				
*Hormonotherapy*				
No	25 (43%)	345 (60%)	1^a^	1^a^^,^^d^
Yes	33 (57%)	230 (40%)	2.0 (1.1–3.4)*	1.9 (1.1–3.4)*

OR=odds ratio;

a Reference category.

b Adjusted for age and stage at diagnosis.

c Adjusted for age, stage at diagnosis, locoregional treatment, oestrogen receptor status and use of hormotherapy.

d Adjusted for age, stage at diagnosis, locoregional treatment, oestrogen receptor status and use of chemotherapy.

* *P*<0.05.

**Table 4:** Use of systemic therapy according to lymph node status, oestrogen receptor status and familial risk

		**Systemic therapy**							
		**Any systemic therapy**		**Anti-oestrogen therapy**		**Chemotherapy**		**Both**	
	**Risk category**	**Yes**	**No**	**Yes**	**No**	**Yes**	**No**	**Yes**	**No**
N0	High risk (*n*=24)	21 (88%)	3 (12%)	16 (67%)	8 (33%)	14 (58%)	10 (42%)	9 (38%)	15 (62%)
	Low risk (*n*=311)	202 (65%)	109 (35%)	120 (39%)	191 (61%)	142 (46%)	169 (54%)	60 (19%)	251 (81%)
	*χ*^2^ test for heterogeneity	0.024	0.007	0.230	0.034				
									
N+	High risk (*n*=31)	29 (94%)	2 (6%)	17 (55%)	14 (45%)	28 (90%)	3 (10%)	16 (52%)	8 (48%)
	Low risk (*n*=248)	231 (93%)	17 (7%)	104 (42%)	144 (58%)	226 (91%)	22 (9%)	99 (40%)	149 (60%)
	*χ*^2^ test for heterogeneity	0.933	0.172	0.882	0.011				
									
ER+	High risk (*n*=43)	40 (93%)	3 (7%)	32 (74%)	11 (26%)	33 (77%)	10 (23%)	25 (58%)	18 (42%)
	Low risk (*n*=367)	289 (79%)	78 (21%)	205 (56%)	162 (44%)	224 (61%)	143 (39%)	140 (38%)	227 (62%)
	*χ*^2^ test for heterogeneity	0.026	0.020	0.044	0.011				
									
ER−	High risk (*n*=11)	9 (82%)	2 (18%)	1 (9%)	10 (91%)	8 (73%)	3 (26%)	0 (0%)	11 (100%)
	Low risk (*n*=143)	115 (80%)	28 (20%)	16 (11%)	127 (89%)	114 (79%)	29 (21%)	15 (10%)	128 (90%)
	*χ*^2^ test for heterogeneity	0.910	0.831	0.528	NA				

N0=no metastasis in axillary lymph nodes; N+=metastasis in axillary lymph nodes; ER+=oestrogen receptors status positive; ER−=oestrogen receptor status negative; NA=not applicable. Note: 19 patients with unknown lymph node status (three high-risk and 16 low-risk patients) and 69 women with unknown oestrogen receptors status (four high-risk and 65 low-risk patients) were not included in this table.

**Table 5:** Impact of familial risk on breast cancer mortality according to age at diagnosis and use of chemotherapy

	***N***	***N* deaths**	**Unadjusted HR**	**Multi adjusted hazard ratio (95% CI)** a
*41–50 years*				
Low risk	441	55	1^b^	1^b^
High risk	48	5	0.9 (0.3–2.1)	1.0 (0.4–2.6)
				
<*41 years*				
Low risk	144	18	1^b^	1^b^
High risk	10	2	2.1 (0.5–9.1)	4.0 (0.8–19.7)
				
*Chemotherapy*				
Low risk	374	54	1^b^	1^b^
High risk	43	5	0.9 (0.4–2.2)	1.0 (0.4–2.6)
				
*No chemotherapy*				
Low risk	201	19	1^b^	1^b^
High risk	15	2	1.5 (0.3–6.4)	2.7 (0.6–12.5)

a Adjusted for socioeconomic status, stage, grade, oestrogen receptor status, locoregional treatment, chemotherapy and hormonotherapy.

b Reference category.
